# Sexual Transcription Differences in *Brachymeria lasus* (Hymenoptera: Chalcididae), a Pupal Parasitoid Species of *Lymantria dispar* (Lepidoptera: Lymantriidae)

**DOI:** 10.3389/fgene.2019.00172

**Published:** 2019-03-05

**Authors:** Peng-Cheng Liu, Shuo Tian, De-Jun Hao

**Affiliations:** ^1^Co-Innovation Center for the Sustainable Forestry in Southern China, Nanjing Forestry University, Nanjing, China; ^2^The College of Forestry, Nanjing Forestry University, Nanjing, China

**Keywords:** sexually dimorphic, *Brachymeria lasus*, transcriptomic analysis, sex determination, venom protein, transient receptor potential channels

## Abstract

Sex differences in gene expression have been extensively documented, but little is known about these differences in parasitoid species that are widely applied to control pests. *Brachymeria lasus* is a solitary parasitoid species and has been evaluated as a potential candidate for release to control *Lymantria dispar*. In this study, gender differences in *B. lasus* were investigated using Illumina-based transcriptomic analysis. The resulting 37,453 unigene annotations provided a large amount of useful data for molecular studies of *B. lasus*. A total of 1416 differentially expressed genes were identified between females and males, and the majority of the sex-biased genes were female biased. Gene Ontology (GO) and Pathway enrichment analyses showed that (1) the functional categories DNA replication, fatty acid biosynthesis, and metabolism were enhanced in females and that (2) the only pathway enriched in males was phototransduction, while the GO subcategories enriched in males were those involved in membrane and ion transport. In addition, thirteen genes involving transient receptor potential (TRP) channels were annotated in *B. lasus*. We further explored and discussed the functions of TRPs in sensory signaling of light and temperature. In general, this study provides new molecular insights into the biological and sexually dimorphic traits of parasitoids, which may improve the application of these insects to the biological control of pests.

## Introduction

Parasitoids are animals that parasitize other organisms (Godfray, 1994). All invertebrate life stages, such as egg, larva or nymph, pupa and adult, can be attacked by oviposition on or in the host or by depositing a larva on or near a host (Boulton et al., 2015). Based on the number of offspring reared in a host, parasitoid wasps are classified as solitary (one parasitoid per host), quasi-gregarious (one parasitoid per host, but hosts are spatially clumped, such as a clutch of eggs on a leaf), or gregarious (multiple parasitoids per host). The vast majority of parasitoids are solitary wasps (Mayhew, 1998). Parasitoids can also be classified as koinobionts (in which hosts continue to develop and grow to some extent) or idiobionts (in which hosts do not grow further). Parasitoid wasps are haplodiploid: males develop from unfertilized eggs and are haploid, while females develop from fertilized eggs and are diploid (Cook, 1993; Heimpel and de Boer, 2008). Parasitoid species (e.g., *Sclerodermus harmandi*, *Trichogramma*) are important insects and have been extensively applied to reduce the population size of pest species (Hassan, 1993; Li, 1994; Terayama, 1999; Zhishan et al., 2003; Parra and Zucchi, 2004; Lim et al., 2006). In addition to having important applications, parasitoid and mutualistic Chalcidoidea, such as jewel (*Nasonia vitripennis)* and fig (*Pleistodontes froggatti*) wasps, have been important study models of behavioral ecology and evolutionary biology for such traits as their sexual dimorphism in longevity, body size, and dispersal (Hamilton, 1967; Charnov, 1982; Yan et al., 1989; Godfray, 1994).

Animals from a broad range of taxa show sex differences, which include behavioral (Breedlove, 1992), physiological (Bardin and Catterall, 1981), and morphological dimorphisms (Darwin, 1871). It is often assumed that the majority of sexually dimorphic traits arise from differences in the expression of genes present in both sexes (Connallon and Knowles, 2005; Rinn and Snyder, 2005). Sex-biased gene expression has been documented in brown algae (Lipinska et al., 2015), birds (Pointer et al., 2013), nematodes (Albritton et al., 2014), *Daphnia pulex* (Eads et al., 2007), and multiple insect species, including *Drosophila* (Jin et al., 2001; Arbeitman et al., 2002; Ranz et al., 2003; Chang et al., 2011), *Anopheles gambiae* (Hahn and Lanzaro, 2005; Marinotti et al., 2006; Baker et al., 2011), *Tribolium castaneum* (Prince et al., 2010), vespid wasps (Hunt and Goodisman, 2010), and *Bemisia tabaci* (Wen et al., 2014). However, few studies of sex differences in gene expression have been done in Hymenoptera insects, and these studies have focussed mainly on social species (e.g., honeybee; Cameron et al., 2013) and model organisms of parasitoids, e.g., jewel wasp *N. vitripennis* (Wang et al., 2015), which is a classic gregarious species. Most species of parasitoid wasps are thought of as solitary species (Mayhew, 1998), but their sexual transcription differences have not been addressed.

Gypsy moth, *Lymantria dispar* is a worldwide pest, and its pupal stage can be parasitized by *Brachymeria lasus*. *B. lasus* is a solitary parasitoid species and has been evaluated as a potential candidate for release to control *L. dispar* (Simser and Coppel, 1980), *Homona magnanima* (Mao and Kunimi, 1991) and *Sylepta derogate* (Kang et al., 2006). In addition, *B. lasus* has a wide host range, including many Lepidoptera species (e.g., *Mythimna separata*, *Hyphantria cunea*, and *Cnaphalocrocis medinalis*) (Habu, 1960). Male and female *B. lasus* differ in many important biological traits, including longevity (Mao and Kunimi, 1994b); development time in the egg, larval and pupal stages (Mao and Kunimi, 1994a); secondary symbionts; and body size (Yan et al., 1989). As *B. lasus* is a classic solitary species with many documented sex differences, though not yet at the gene expression level, it was used as the experimental material in this study. To reveal *B. lasus* sex differences at the transcriptional level, we carried out an Illumina-based transcriptomic analysis. This study attempted to provide comprehensive insight into the sexually dimorphic traits of parasitoid wasps at the transcriptome level to improve our understanding of other biological traits with the aim of improving the application of parasitoids to the biological control of pest species.

## Materials and Methods

### Insect Cultures

In northern China, in addition to *L. dispar*, *B. lasus* is also an important pupal parasitoid of *H. cunea*, for which the parasitism ratio is approximately 1.06–3.39% in the field (Yang et al., 2001). To acquire *B. lasus* adults, we collected the pupae of *H. cunea*, which may be parasitized by *B. lasus* and other parasitoid species (e.g., *Coccygomimus disparis* Viereck; *Chouioia cunea* Yang) from a field in Xuzhou City, Jiangsu Province, China, in March 2016. After collection, we isolated the pupae individually in polyethylene tubes (height: 7.5 cm; diameter: 1 cm) whose openings were covered by a cotton ball and incubated them at a temperature of 28 ± 0.5°C, a relative humidity (RH) of 70 ± 5% and a photoperiod of 14 L:10 D. We observed and selected *B. lasus* after adult eclosion.

### Transcriptomic Analyses

For the transcriptomic experiment, only 1-day-old *B. lasus* adults were selected, and the sex was determined under a microscope (Leica M205A, Germany). Then, five adults of the same sex were pooled into a plastic tube (1.5 ml), snap-frozen in liquid nitrogen, and transferred to a –80°C freezer for long-term storage. RNA from each sample group (whole bodies of female and male adults) was extracted with TRIzol reagent (Invitrogen; United States). Each group had three replicates. The quality of the isolated RNA was assessed using a NanoDrop (Thermo Fisher Scientific NanoDrop 2000, United States), and the A260/280 values were all above 2.0. A total of 3 μg total RNA from each sample was converted into cDNA using the NEBNext^®^ Ultra^TM^ RNA Library Prep Kit for Illumina^®^ (NEB, United States). In total, six cDNA libraries were constructed and subsequently sequenced with the Illumina HiSeq 2000 platform by Beijing Biomarker Technologies Co., Ltd, resulting in raw reads. Raw sequence data generated were deposited into Sequence Read Archive (SRA) database of NCBI with the accession no. PRJNA513855. Clean reads were obtained by removing reads containing adapter, poly-N reads and low-quality reads from the raw data using FASTX-Toolkit^1^, and these clean reads were used for further analysis. Then, transcriptome assembly was performed using Trinity (v2.5.1) with the default parameters (Grabherr et al., 2011). For functional annotation, pooled assembled unigenes were searched using BLASTX (v2.2.31) against five public databases, Clusters of Orthologous Groups (COG), Swiss-Prot, NCBI non-redundant protein sequences (nr), KEGG Ortholog database (KO) and GO, with an *E*-value cutoff of 10^-5^. Using our assembled transcriptome as a reference, we identified putative genes expressed in males and females by RSEM (Li and Dewey, 2011), using the reads per kb per million reads (RPKM) method. Genes with at least 2-fold changes (i.e., log_2_∣FC∣≥ 1) and a false discovery rate [FDR] < 0.01 as found by DESeq R package (1.10.1) were considered differentially expressed. The GOseq R package (Young et al., 2010) and KOBAS software (Mao et al., 2005) were used to implement the statistical enrichment of differentially expressed genes (DEGs) in the GO and KEGG pathways, respectively, and an adjusted Q-value <0.05 was chosen as the significance cutoff.

### Validation by mRNA Expression and Behavior

Based on transcriptomic data, a gene of transient receptor potential (*trp*) involved in the phototransduction pathway enriched only in males (ko: 04745; Supplementary Figure S1-d), *trp* (Leung et al., 2000), was down-regulated in females, which may lead to a reduction in light response (Leung et al., 2000; Popescu et al., 2006). Therefore, we checked this result at the mRNA expression and behavioral levels.

#### Quantitative Real-Time PCR (qRT-PCR) Analysis

Total RNA was extracted from the whole bodies of five female or five male adults reared on the pupae of *H. cunea* using TRIzol (Invitrogen; United States) according to the manufacturer’s protocols, then resuspended in nuclease-free water. Finally, the RNA concentration was measured using a NanoDrop (Thermo Fisher Scientific NanoDrop 2000; United States). Each group have four replicates. Approximately 0.5 mg of total RNA was used as template to synthesize the first-strand cDNA using a PrimeScript RT Reagent Kit (TaKaRa; Japan) following the manufacturer’s protocols. The resultant cDNA was diluted to 0.1 mg/ml for further qRT-PCR analysis (ABI StepOne Plus; United States) using SYBR Green Real-Time PCR Master Mix (TaKaRa; Japan). Primers (Supplementary Table S1) for *trp* gene were designed using Primer Express 2.0 software. The cycling parameters were 95°C for 30 s followed by 40 cycles of 95°C for 5 s and 62°C for 34 s, ending with a melting curve analysis (65 to 95°C in increments of 0.5°C every 5 s) to check for nonspecific product amplification. Relative gene expression was calculated by the 2^-ΔΔCt^ method using the housekeeping gene GAPDH as a reference to eliminate sample-to-sample variations in the initial cDNA samples.

#### Phototaxis Assays

A glass Y-maze (main arm: 12 cm; two side arms: 5 cm; inner diameter: 1.5 cm; angle between two side arms: 75°) was used for phototaxis assays in a completely dark room (<10 lux, measured by illuminometer, LX-9621, China) at a temperature of 22–26°C. One 1-day-old *B. lasus* adult (female or male) began the trial in a tube at the base of the apparatus and faced a choice between two tubes, one of which was dark and the other of which was lighted with a 40-watt bulb (approximately 600 lux). After 1 min, the choice was recorded. The sample sizes of the male and female groups were 18 and 24, respectively. After each test, the Y-maze was washed and dried, and the two side arms were changed for the new test.

### Statistical Analysis

Prior to analysis, the raw data were tested for normality and homogeneity of variances with the Kolmogorov-Smirnov test and Levene’s test, respectively, and the data were log-transformed if necessary. The qRT-PCR data comparing gene expression in females and males were analyzed with the independent *t*-test. In phototaxis assays, the preferences for light and dark were analyzed using sign tests, and the differences in female and male phototaxis were analyzed by the chi-square test. All analyses were performed using SPSS v.20 (IBM SPSS, Armonk, NY, United States).

## Results and Discussion

Sexual dimorphism is the condition where the two sexes of the same species exhibit different characteristics (e.g., size, color, behavior) beyond the differences in their sexual organs (Bonduriansky, 2007). Most sexually dimorphic traits are often assumed to arise from differences in the expression of genes present in both sexes (Connallon and Knowles, 2005; Rinn and Snyder, 2005). To reveal *B. lasus* sex differences at the transcriptional level, we carried out an Illumina-based transcriptomic analysis.

### Transcriptome Sequencing, Read Assembly and Annotation

All high-quality reads (101,945,678) from the six samples were pooled and assembled by using Trinity with the default parameters, and a total of 254,656 transcripts with lengths longer than 200 bp were generated. The N50 size was 2706 bp with 57,605 sequences longer than 1 kb. We chose the longest isoform of each gene to construct the unigene set. After isoforms were considered, these assembled transcripts were predicted to be produced from a total of 164,709 unigenes. The N50 size of the unigenes was approximately 814 bp, and their mean length was 572.08 bp (Supplementary Table S2). For annotation, the pooled assembled unigenes were searched using blastx against five public databases with an *E*-value cutoff of 10^-5^. A total of 37,453 unigenes were successfully annotated, as shown in Table 1, including 17,248 genes in GO, 13,491 genes in COG, 35,427 genes in nr, 18,195 genes in Swiss-Prot, and 15,133 genes in KEGG.

**Table 1:** Annotation of a pooled assembly including both male and female *B. lasus* transcriptomes.

Annotation database	Annotated unigenes	Number of DEGs
COG	13,491	420
GO	17,248	442
KEGG	15,133	396
Swiss-Prot	18,195	613
nr	35,427	1024
Total	37,453	1416


In the GO analysis, 17,248 unigenes were successfully annotated and classified into three major GO categories: molecular function (MF), cell component (CC), and biological processes (BP), then assigned to 56 subcategories based on GO level 2. The dominant subcategories for the classified genes were catalytic activity and binding for the MF category; cell and cell part for the CC category; and metabolic process, cellular process, and single-organism process for the BP category (Supplementary Table S3). A total of 15,133 KEGG-annotated unigenes were classified into 190 pathways (>10 associated unigenes). Among these pathways, the ten most highly represented were ribosome, carbon metabolism, protein processing in endoplasmic reticulum, oxidative phosphorylation, biosynthesis of amino acids, spliceosome, RNA transport, purine metabolism, peroxisome, and ubiquitin mediated proteolysis (Supplementary Table S4).

### Sex-Biased Genes

Although in most species the male and female genomes differ by a few genes located on sex-specific chromosomes (such as the Y chromosome of mammals), the vast majority of sexually dimorphic traits result from the differential expression of genes that are present in both sexes (Connallon and Knowles, 2005; Rinn and Snyder, 2005; Ellegren and Parsch, 2007), and this is especially true in hymenopteran insects. Because sex determination in hymenopteran species is haplodiploid, females and males are nearly identical genetically (Ellegren and Parsch, 2007). Such DEGs include those that are expressed exclusively in one sex (sex-specific expression) and those that are expressed in both sexes but at a higher level in one sex (sex-biased expression). These sex-biased genes can be further separated into male-biased and female-biased genes, depending on which sex shows higher expression. Genes with equal expression in the two sexes are referred to as unbiased (Ellegren and Parsch, 2007).

Using our assembled transcriptome as a reference, we identified putative genes expressed in males and females using the RPKM method, and genes with at least 2-fold changes and FDR < 0.01 were defined as DEGs. By comparing female and male transcriptomes, 1416 DEGs were found in *B. lasus*, of which 442 genes were annotated in GO, 420 in COG, 1024 in nr, 613 in Swiss-Prot, and 396 in KEGG (Table 1). Among these DEGs, 986 were up-regulated in females and 430 were up-regulated in males (Supplementary Table S5).

#### GO Enrichment Analyses

**FIGURE 1:**
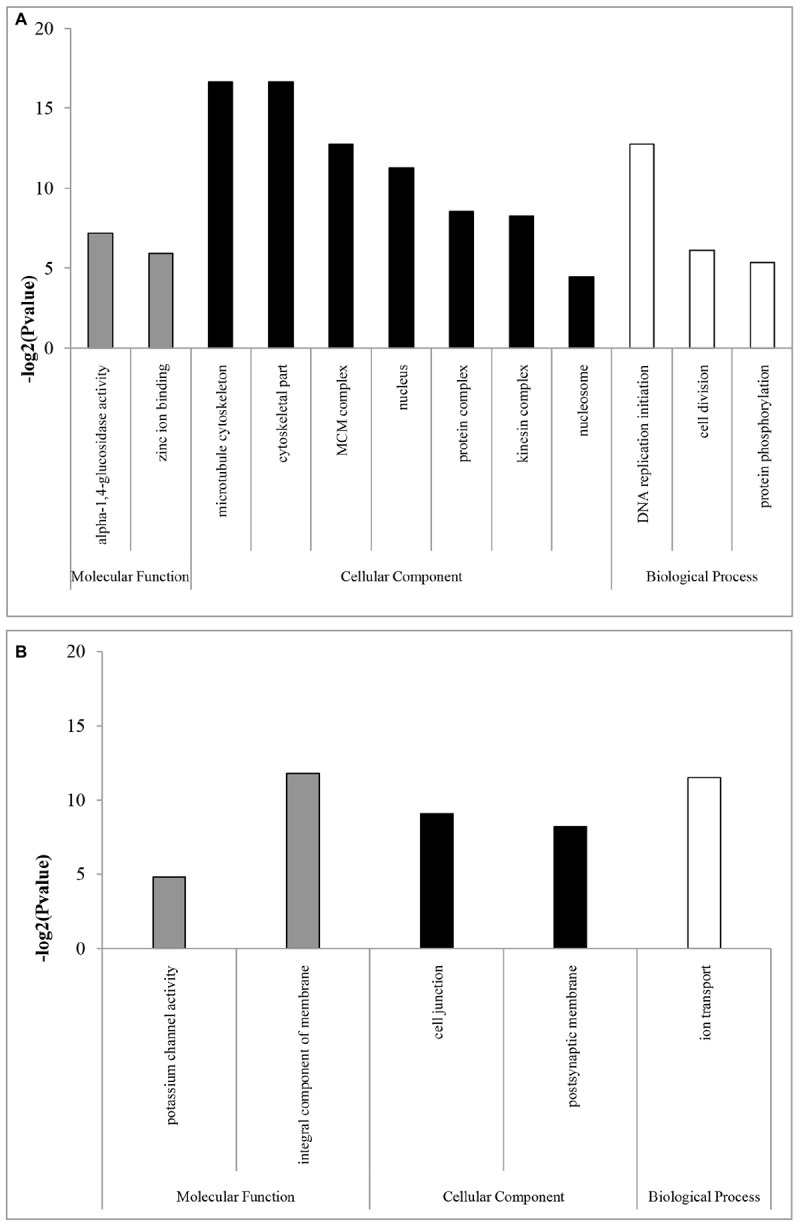
GO enrichment analysis of **(A)** female- and **(B)** male-biased genes. GOSeq explicitly takes into account gene selection bias due to differences in gene length and thus the numbers of overlapping sequencing reads. GOSeq was used for the GO enrichment analysis, and an adjusted Q-value <0.05 was chosen as the significance cutoff.

In the GO enrichment analyses, 12 and five subcategories were enriched in females and males, respectively. In females, the enriched subcategories were microtubule cytoskeleton, cytoskeletal part, MCM complex, nucleus, protein complex, kinesin complex, and nucleosome for the CC category; DNA replication initiation, cell division and protein phosphorylation for the BP category; and alpha-1,4-glucosidase activity and zinc ion binding for the MF category (Figure 1A). These results showed that, consistent with the results in flies, mosquitoes, and *Daphnia* (Ranz et al., 2003; Hahn and Lanzaro, 2005; Eads et al., 2007), including Hymenoptera insects of *Nasonia* (Wang et al., 2015), most categories were related to DNA replication, which are probably expressed to produce eggs in females (Spradling, 1993; Parisi et al., 2004). The over-representation of transcripts from genes required for DNA replication may be required for nurse cell polyploidization or for the rapid division of embryonic cells, which rely on maternally deposited gene products (Spradling, 1993; Parisi et al., 2004).

In males, the enriched subcategories were integral component of membrane, cell junction, and postsynaptic membrane for the CC category; ion transport for the BP category; and potassium channel activity for the MF category (Figure 1B), consistent with a study in *D. melanogaster* (Parisi et al., 2004), which may be mainly related to spermatogenesis (Fuller, 1993). For example, the enriched subcategories associated with membranes were likely due to the requirements of the sperm axoneme structure (Parisi et al., 2004). However, in parasitoids of *N. vitripennis* species, highly enriched subcategories in males are related to sex-pheromone synthetic enzymes (Wang et al., 2015). Those differences might be likely to contribute by their difference in sexual maturity period. Sexual maturity in many gregarious and quasi-gregarious males (e.g., *N. vitripennis*) happens before eclosion, and these males can immediately mate with females after eclosion and near the emergence site (Boulton et al., 2015), while solitary *B. lasus* have mating ability for some days after eclosion (Yan et al., 1989).

#### KEGG Pathway Enrichment Analyses

Consistent with the results of GO enrichment in females, pathway enrichment tests revealed that DNA replication (ko: 03030; Supplementary Figure S1-a) was enriched in *B. lasus* females. The functional categories enriched in females also included fatty acid biosynthesis (ko: 00061; Supplementary Figure S1-b) and metabolism (ko01212; Supplementary Figure S1-c). The fatty acid synthase gene (FASN), which encoded the enzyme catalyzing fatty acid synthesis (Jayakumar et al., 1994, 1995; Persson et al., 2008), was probably crucial for egg yolk production and thus female fecundity. In some insects, for example yellow fever mosquito *Aedes aegypti*, brown planthopper *Nilaparvata lugens*) (Alabaster et al., 2011; Li et al., 2016), when *FAS* expression decreases in females, the number of oviposited eggs significantly decreases.

We found that only the phototransduction-fly pathway (ko: 04745; Supplementary Figure S1-d) was enriched in males, which is associated with perception of light signals (Leung et al., 2000). Its potential functions are discussed below.

### Annotated Genes Involved in Venom Proteins

In terms of biological control, parasitoid species have been extensively applied for reducing pest species population sizes (Hassan, 1993; Li, 1994; Terayama, 1999; Zhishan et al., 2003; Parra and Zucchi, 2004; Lim et al., 2006) because parasitoids can propagate on or in other arthropods. The venom of parasitoid wasps, which is injected into a host by females before or at oviposition, is important for the successful development of the progeny. Parasitoid venoms have diverse physiological effects on hosts, including developmental arrest; alteration in growth and physiology; suppression of immune responses; induction of paralysis, oncosis, or apoptosis; and alteration of host behavior (Edwards et al., 2006; Price et al., 2009; Tian et al., 2010; Kryukova et al., 2011). In total, three female-biased genes (c100635.graph_c0, c101314.graph_c0, c101670.graph_c0; Supplementary Table S5) in this study were annotated for venom proteins, which were related to known insect venoms from *N. vitripennis* and belonged to previously known insect venom families, such as serine proteases (Graaf et al., 2010; Werren et al., 2010). Despite the large diversity of parasitoid wasp species, only a small number of venom proteins have been described from wasps. A wealth of unexplored biomolecules is present in parasitoid venoms; these proteins are of value in basic evolutionary studies, venom biology, host-parasite interactions, and the study of the evolution of life strategies, and they may potentially contain components that could be used in biological control and pharmacology (Moreau and Asgari, 2015).

### Annotation of Genes in the TRP Channel Family and Function Validation

Transient Receptor Potential channels are cation channels that are mainly considered as unique polymodal cell sensors; TRPs can be subdivided into six main subfamilies: the TRPC (canonical), TRPV (vanilloid), TRPM (melastatin), TRPP (polycystin), TRPML (mucolipin), and TRPA (ankyrin) groups (Gees et al., 2010). Functionally, TRP channels cause cell depolarization when activated, which may trigger many voltage-dependent ion channels. Upon stimulation, Ca^2+^-permeable TRP channels generate changes in the intracellular Ca^2+^ concentration, [Ca^2+^]_i_, due to Ca^2+^ entry via the plasma membrane. However, evidence is increasing that TRP channels are also located in intracellular organelles and serve as intracellular Ca^2+^ release channels (Berridge et al., 2000; Bootman et al., 2001; Gees et al., 2010). TRP channels in *Drosophila* are involved in the perception of sensory signals such as light, temperature, humidity, pheromones, sound, and touch (Lin et al., 2005). In our study, we found 13 TRP channel genes in *B. lasus*; *Nasonia* and honey bee contain 12 and 11 genes, respectively, indicating that the number of *trp* channels seems to be well conserved in Hymenoptera (Werren et al., 2010). Of the TRP channel genes in *B. lasus*, most belong to two subfamilies: TRPC and TRPA (Table 2).

**Table 2:** TRP channel genes in the *B. lasus* transcriptome.

Gene name	Subfamily	*Drosophila* orthologue name	Function in *Drosophila*	Comparative analyses with RNAseq data
c103240.graph_c0	TRPC	*trp*	phototransduction	up
c107438.graph_c0		*trp* gamma	phototransduction	normal
c107438.graph_c1		*trp* gamma	phototransduction	normal
c87378.graph_c0		*trp* gamma	phototransduction	normal
c107458.graph_c0	TRPM	*trpm*	unknown	normal
c107458.graph_c1		*trpm*	unknown	normal
c103139.graph_c0	TRPA	pyrexia	geotaxis	normal
c106854.graph_c0		pyrexia	geotaxis	normal
c107721.graph_c1		pyrexia	geotaxis	normal
c108434.graph_c0		pyrexia	geotaxis	normal
c89491.graph_c0		pyrexia	geotaxis	up
c106747.graph_c0		painless	nociception	normal
c108178.graph_c0	TRPML	*trpml*	TRPML	normal


**FIGURE 2:**
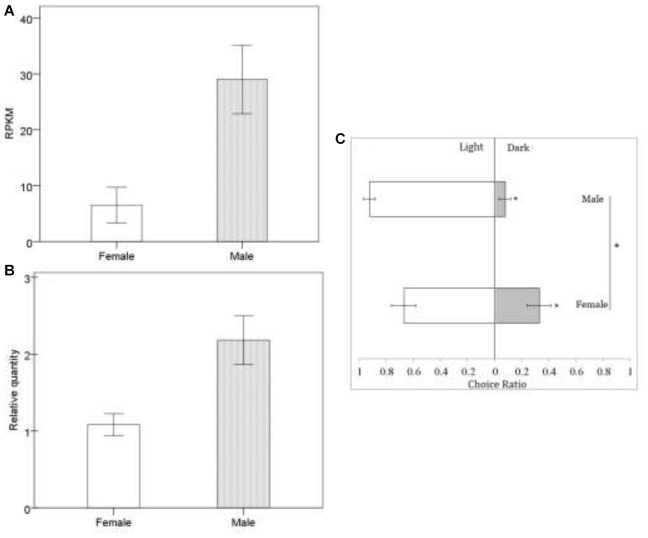
Sexual difference in response to light at mRNA level **(A,B)** and behavioral level **(C)**. In transcriptomic data, we identified putative genes expressed using the reads per kb per million reads (RPKM) method. Quantitative real-time PCR (qRT-PCR) analysis was used to calculated the relative gene expression to further check the transcriptomic data, in which the differences in female and male were analyzed by the independent *t*-test. There was a highly significant correlation co-efficient of 0.885 between transcriptomic data and qRT-PCR data. Behavioral responses of *Brachymeria lasus* adults to dark or light were tested with phototaxis assays. The differences in female (*n* = 24) and male (*n* = 18) phototaxis were analyzed by the chi-square test. ^∗^indicates *p* < 0.05. The error bars indicate standard errors.

In *Drosophila*, TRPC plays an important role in the perception of light signals, i.e., the phototransduction pathway (Leung et al., 2000) (ko: 04745; Supplementary Figure S1-d), which was enriched in *B. lasus* male adults. In *Drosophila*, a number of genes in the visual signal transduction pathway have been characterized, with functions including rhodopsin activation, phosphoinoside signaling, and the opening of TRP and TRPL channels (Wolff and Ready, 1993; Zuker, 1996; Leung et al., 2000; Wang and Montell, 2007). Our transcriptional analyses (Figure 2A: FDR < 0.01, log_2_ FC = 1.62) and q-PCR results (Figure 2B: *t* = -3.169, *df* = 6, *p* = 0.019), showed that the gene corresponding to *trp* (c103240.graph_c0) was more highly expressed in *B. lasus* males, consistent with the phototaxis test. Although both females and males tended to move toward light (Figure 2C: female, *Z* = -1.34, *p* < 0.05; male, *Z* = -1.6, *p* < 0.05), the tendency to prefer light was significantly influenced by sex in adults (Figure 2C. *χ*^2^ = 4.17, *df* = 1, *p* < 0.05), males more preferring to move to light. This result is similar to the results of research on *trp* mutants in *Drosophila*, which had altered phenotypes, including a reduction in light response (Leung et al., 2000; Popescu et al., 2006). Female reduction in light response might be due to their long periods living in the dark to search for hosts and lay offspring into them, as most host species (e.g., pupae of *L. dispar* or *H. cunea*) hide in dark environments, such as the litter horizon (Yan et al., 1989; Yang et al., 2001). Surprisingly, five members of the TRPA subfamily, which is involved in sensing environmental temperature, were annotated in our study. Animals must maintain thermal homeostasis and avoid prolonged contact with harmfully hot or cold objects (Caterina, 2007; Karashima et al., 2009). Unlike most parasitoid species, which overwinter in their hosts as eggs or larvae, *B. lasus* lives through the winter in its adult stage (Yan et al., 1989). Thus, TRPA may be essential for *B. lasus* adults, allowing them to sense harmful cold during winter. In addition, intraspecific aggregations in *B. lasus* have been observed in previous research, and an active component that elicited the aggregation response was isolated and identified as 3-hexanone (Mohamed and Coppel, 1987). The effects of aggregation behavior include mating, host attack, defense, and thermoregulation, and in this species, a previous study suggested that aggregation resulted from an increase in reproductive success by increasing the probability of mate location, as well as offering the possibility of mate choice (Mohamed and Coppel, 1987). However, combining the above results, adults may also aggregate at a site for purposes of thermoregulation, especially in winter, in response to cold. Further studies are required to elucidate the nature of this cue.

## Conclusion

*Brachymeria lasus* is a solitary parasitoid species and has been evaluated as a potential candidate for release to control *L. dispar*. Whereas previous studies have focussed on the application of parasitoids and their sex differences in phenotypes, this study focussed mainly on sex differences in gene expression. *Brachymeria lasus* as a representative of solitary species was studied, which enriched our understanding of sexual transcription differences in parasitoid wasps, especially solitary species. Here, we performed transcriptome assembly using the Trinity program, which provided a large amount of useful information for molecular studies of *B. lasus*, including venom protein and perception of sensory signals. In addition to sex-biased genes, epigenetic processes, such as DNA methylation, are known to play important roles in differentiating phenotype and have been widely studied in Hymenopteran insects, for example, female morphs (queens and workers) in the honeybee, *Apis mellifera* (Kucharski et al., 2008; Lyko et al., 2010), although these processes do not appear to be in *Nasonia* (Wang et al., 2015). More future research will be conducted to better understand the molecular mechanisms underlying the biological traits of sex differences in *B. lasus* and to better apply this parasitoid to the biological control of pests.

## Data Availability

Publicly available datasets were analyzed in this study. This data can be found here: https://dataview.ncbi.nlm.nih.gov/object/PRJNA513855.

## Ethics statement

There was no requirement to seek ethical approval to carry out the work described above. However, the use of insects in the above experiments was kept to a minimum.

## Author Contributions

P-CL conceived and designed the experiments. P-CL and ST performed the experiments. P-CL and D-JH wrote the manuscript. All the authors reviewed the manuscript.

## Conflict of Interest Statement

The authors declare that the research was conducted in the absence of any commercial or financial relationships that could be construed as a potential conflict of interest.

## Footnotes

1 http://hannonlab.cshl.edu/fastx_toolkit/

## References

[B1] Alabaster A. Isoe J. Zhou G. Lee A. Murphy A. Day W. A. (2011). Deficiencies in acetyl-CoA carboxylase and fatty acid synthase 1 differentially affect eggshell formation and blood meal digestion in *Aedes aegypti*. *Insect Biochem. Mol. Biol.* 41 946–955. 10.1016/j.ibmb.2011.09.004 21971482 PMC3210400

[B2] Albritton S. E. Kranz A. L. Rao P. Kramer M. Dieterich C. Ercan S. (2014). Sex-biased gene expression and evolution of the x chromosome in nematodes. *Genetics* 197 865–883. 10.1534/genetics.114.163311 24793291 PMC4096367

[B3] Arbeitman M. N. Furlong E. E. Imam F. Johnson E. Null B. H. Baker B. S. (2002). Gene expression during the life cycle of *Drosophila melanogaster*. *Science* 297 2270–2275. 10.1126/science.1072152 12351791

[B4] Baker D. A. Nolan T. Fischer B. Pinder A. Crisanti A. Russell S. (2011). A comprehensive gene expression atlas of sex- and tissue-specificity in the malaria vector. *Anopheles gambiae. BMC Genomics* 12:296. 10.1186/1471-2164-12-296 21649883 PMC3129592

[B5] Bardin C. W. Catterall J. F. (1981). Testosterone: a major determinant of extragenital sexual dimorphism. *Science* 211 1285–1294. 10.1126/science.7010603 7010603

[B6] Berridge M. J. Lipp P. Bootman M. D. (2000). The versatility and universality of calcium signalling. *Nat. Rev. Mol. Cell Biol.* 1 11–21. 10.1038/35036035 11413485

[B7] Bonduriansky R. (2007). The evolution of condition-dependent sexual dimorphism. *Am. Nat.* 169 9–19. 17206580 10.1086/510214

[B8] Bootman M. D. Collins T. J. Peppiatt C. M. Prothero L. S. MacKenzie L. De Smet P. (2001). Calcium signalling-an overview. *Semin. Cell Dev. Biol.* 12 3–10. 10.1006/scdb.2000.0211 11162741

[B9] Boulton R. A. Collins L. A. Shuker D. M. (2015). Beyond sex allocation: the role of mating systems in sexual selection in parasitoid wasps. *Biol. Rev.* 90 599–627. 10.1111/brv.12126 24981603 PMC4409842

[B10] Breedlove S. M. (1992). Sexual dimorphism in the vertebrate nervous-system. *J. Neurosci.* 12 4133–4142. 10.1523/JNEUROSCI.12-11-04133.1992 1432094 PMC6575986

[B11] Cameron R. C. Duncan E. J. Dearden P. K. (2013). Biased gene expression in early honeybee larval development. *BMC Genomics* 14:903. 10.1186/1471-2164-14-903 24350621 PMC3878232

[B12] Caterina M. J. (2007). Transient receptor potential ion channels as participants in thermosensation and thermoregulation. *Am. J. Physiol. Regul. Integr. Comp. Physiol.* 292 R64–R76. 10.1152/ajpregu.00446.2006 16973931

[B13] Chang P. L. Dunham J. P. Nuzhdin S. V. Arbeitman M. N. (2011). Somatic sex specific transcriptome differences in Drosophila revealed by whole transcriptome sequencing. *BMC Genomics* 12:364. 10.1186/1471-2164-12-364 21756339 PMC3152543

[B14] Charnov E. L. (1982). *The Theory of Sex Allocation.* Princeton: Princeton University Press. 7144766

[B15] Connallon T. Knowles L. L. (2005). Intergenomic conflict revealed by patterns of sex-biased gene expression. *Trends Genet.* 21 495–499. 10.1016/j.tig.2005.07.006 16039005

[B16] Cook J. M. (1993). Sex determination in the hymenoptera-a review of models and evidence. *Heredity* 71 421–435. 10.1038/hdy.1993.157

[B17] Darwin C. R. (1871). *The Descent of Man, and Selection in Relation to Sex*, 2nd Edn London: John Murray.

[B18] Eads B. D. Colbourne J. K. Bohuski E. Andrews J. (2007). Profiling sex-biased gene expression during parthenogenetic reproduction in *Daphnia pulex*. *BMC Genomics* 8:464. 10.1186/1471-2164-8-464 18088424 PMC2245944

[B19] Edwards J. P. Bell H. A. Audsley N. Marris G. C. Kirkbride-Smith A. Bryning G. (2006). The ectoparasitic wasp *Eldophus pennicornis* (Hymenoptera: Eulophiclae) uses instar-specific endocrine disruption strategies to suppress the development of its host *Lacanobia oleracea* (Lepidoptera: Noctuidae). *J. Insect Physiol.* 52 1153–1162. 10.1016/j.jinsphys.2006.08.003 17064726

[B20] Ellegren H. Parsch J. (2007). The evolution of sex-biased genes and sex-biased gene expression. *Nat. Rev. Genet.* 8:689. 10.1038/nrg2167 17680007

[B21] Fuller M. T. (1993). “Spermatogenesis,” in *The Development of Drosophila*, eds Bate M. Martinez-Arias A. (Cold Sping Harbor, NY: Cold Sping Harbor Laboratory Press), 71–148.

[B22] Gees M. Colsoul B. Nilius B. (2010). The role of transient receptor potential cation channels in Ca2+ Signaling. *Cold Spring Harb. Perspect. Biol.* 2:a003962. 10.1101/cshperspect.a003962 20861159 PMC2944357

[B23] Godfray H. C. J. (1994). *Parasitoids: Behavioural and Evolutionary Ecology.* Princeton: Princeton University Press.

[B24] Graaf D. C. D. Aerts M. Brunain M. Desjardins C. A. Jacobs F. J. Werren J. H. (2010). Insights into the venom composition of the ectoparasitoid wasp *Nasonia vitripennis* from bioinformatic and proteomic studies. (special issue: the *Nasonia* genome.). *Insect Mol. Biol.* 19 11–26. 10.1111/j.1365-2583.2009.00914.x 20167014 PMC3544295

[B25] Grabherr M. G. Haas B. J. Yassour M. Levin J. Z. Thompson D. A. Amit I. (2011). Full-length transcriptome assembly from RNA-seq data without a reference genome. *Nat. Biotechnol.* 29 644–652. 10.1038/nbt.1883 21572440 PMC3571712

[B26] Habu A. (1960). A revision of the Chalcididae (Hymenoptera) of Japan, with descriptions of sixteen new species. *Bull. Natl. Inst. Agric. Sci.* 11 131–363.

[B27] Hahn M. W. Lanzaro G. C. (2005). Female-biased gene expression in the malaria mosquito *Anopheles gambiae*. *Curr. Biol.* 15 192–193. 10.1016/j.cub.2005.03.005 15797007

[B28] Hamilton W. D. (1967). Extraordinary sex ratios. *Science* 156 477–488. 10.1126/science.156.3774.477 6021675

[B29] Hassan S. A. (1993). The mass rearing and utilization of *Trichogramma* to control lepidopterous pests: achievements and outlook. *Pest Manage. Sci.* 37 387–391. 10.1002/ps.2780370412

[B30] Heimpel G. E. de Boer J. G. (2008). Sex determination in the Hymenoptera. *Ann. Rev. Entomol.* 53 209–230. 10.1146/annurev.ento.53.103106.093441 17803453

[B31] Hunt B. G. Goodisman M. A. (2010). Evolutionary variation in gene expression is associated with dimorphism in eusocial *vespid wasps*. *Insect Mol. Biol.* 19 641–652. 10.1111/j.1365-2583.2010.01021.x 20546040

[B32] Jayakumar A. Chirala S. S. Chinault A. C. Baldini A. Abu-Elheiga L. Wakil S. J. (1994). Isolation and chromosomal mapping of genomic clones encoding the human fatty acid synthase gene. *Genomics* 23 420–424. 10.1006/geno.1994.1518 7835891

[B33] Jayakumar A. Tai M. H. Huang W. Y. Al-Feel W. Hsu M. Abu-Elheiga L. (1995). Human fatty acid synthase: properties and molecular cloning. *Proc. Natl. Acad. Sci. U.S.A.* 92 8695–8699. 10.1073/pnas.92.19.8695 7567999 PMC41033

[B34] Jin W. Riley R. M. Wolfinger R. D. White K. P. Passadorgurgel G. Gibson G. (2001). The contributions of sex, genotype and age to transcriptional variance in *Drosophila melanogaster*. *Nat. Genet.* 29:389. 10.1038/ng766 11726925

[B35] Kang X. X. Chen J. Wang C. C. Yang Y. Z. (2006). Identification and behaviors of parasitoids of *Sylepta derogata* in the Yangtze River and Huihe Valley. *Chin. Bull. Entomol.* 35 241–245.

[B36] Karashima Y. Talavera K. Everaerts W. Janssens A. Kwan K. Y. Vennekens R. (2009). Trpa1 acts as a cold sensor in vitro and in vivo. *Proc. Natl. Acad. Sci. U.S.A.* 106 1273–1278. 10.1073/pnas.0808487106 19144922 PMC2633575

[B37] Kryukova N. Dubovskiy I. Chertkova E. Vorontsova Y. Slepneva I. Glupov V. (2011). The effect of Habrobracon hebetor venom on the activity of the prophenoloxidase system, the generation of reactive oxygen species and encapsulation in the haemolymph of *Galleria mellonella* larvae. *J. Insect Physiol.* 57 769–800. 10.1016/j.jinsphys.2011.03.008 21419772

[B38] Kucharski R. Maleszka J. Foret S. Maleszka R. (2008). Nutritional control of reproductive status in honeybees via DNA methylation. *Science* 319 1827–1830. 10.1126/science.1153069 18339900

[B39] Leung H. T. Geng C. Pak W. L. (2000). Phenotypes of trpl mutants and interactions between the transient receptor potential (TRP) and TRP-like channels in Drosophila. *J. Neurosci.* 20 6797–6803. 10.1523/JNEUROSCI.20-18-06797.2000 10995823 PMC6772831

[B40] Li B. Dewey C. N. (2011). RSEM: accurate transcript quantification from RNA-Seq data with or without a reference genome. *BMC Bioinformatics* 12:323. 10.1186/1471-2105-12-323 21816040 PMC3163565

[B41] Li L. (1994). “Worldwide use of Trichogramma for biological control on different crops: a survey,” in *Biological Control with Egg Parasitoids*, eds Wajnberg E. Hassan S. A. (Wallingford: Cab International).

[B42] Li L. Jiang Y. Liu Z. You L. Wu Y. Xu B. (2016). Jinggangmycin increases fecundity of the brown planthopper, *Nilaparvata lugens* (Stål) via fatty acid synthase gene expression. *J. Proteomics* 130 140–149. 10.1016/j.jprot.2015.09.022 26388431

[B43] Lim J. O. Lyu D. P. Choi G. S. Jeong Y. J. Shin S. C. Lee S. H. (2006). A taxonomie note on *Sclerodermas harmandi*, ectoparasite of stem and wood boring insect larvae (Hymenoptera: Chrysidoidea’-Bethylidae) in South Korea. *J. Asia Pac. Entomol.* 9 115–119. 10.1016/S1226-8615(08)60282-4

[B44] Lin H. Mann K. J. Starostina E. Kinser R. D. Pikielny C. W. (2005). A Drosophila DEG/ENaC channel subunit is required for male response to female pheromones. *Proc. Natl. Acad. Sci. U.S.A.* 102 12831–12836. 10.1073/pnas.0506420102 16129837 PMC1200314

[B45] Lipinska A. Cormier A. Luthringer R. Peters A. F. Corre E. Gachon C. M. (2015). Sexual dimorphism and the evolution of sex-biased gene expression in the brown alga ectocarpus. *Mol. Biol. Evol.* 32 1581–1597. 10.1093/molbev/msv049 25725430

[B46] Lyko F. Foret S. Kucharski R. Wolf S. Falckenhayn C. Maleszka R. (2010). The honey bee epigenomes: differential methylation of brain DNA in queens and workers. *PLoS Biol.* 8:e1000506. 10.1371/journal.pbio.1000506 21072239 PMC2970541

[B47] Mao H. Kunimi Y. (1991). Pupal mortality of the oriental tea tortrix, *Homona magnanima* Diakonoff (Lepidoptera: Tortricidae), caused by parasitoids and pathogens. *Jpn. J. Appl. Entomol. Zool.* 35 241–245. 10.1303/jjaez.35.241

[B48] Mao H. Kunimi Y. (1994a). Effects of temperature on the development and parasitism of *Brachymeria lasus*, a pupal parasitoid of *Homona magnanima*. *Entomol. Exp. Appl.* 71 87–90. 10.1111/j.1570-7458.1994.tb01773.x

[B49] Mao H. Kunimi Y. (1994b). Longevity and fecundity of *Brachymeria lasus* (Walker) (Hymenoptera: Chalcididae), a pupal parasitoid of the Oriental tea tortrix, *Homona magnanima* Diakonoff (Lepidoptera: Tortricidae) under laboratory conditions. *Appl. Entomol. Zool.* 29 237–243. 10.1303/aez.k29.237

[B50] Mao X. Cai T. Olyarchuk J. G. Wei L. (2005). Automated genome annotation and pathway identification using the KEGG Orthology (KO) as a controlled vocabulary. *Bioinformatics* 21 3787–3793. 10.1093/bioinformatics/bti430 15817693

[B51] Marinotti O. Calvo E. Nguyen Q. K. Dissanayake S. Ribeiro J. M. James A. A. (2006). Genome-wide analysis of gene expression in adult *Anopheles gambiae*. *Insect Mol. Biol.* 15 1–12. 10.1111/j.1365-2583.2006.00610.x 16469063

[B52] Mayhew P. J. (1998). The life-histories of parasitoid wasps developing in small gregarious broods. *Neth. J. Zool.* 48 225–240. 10.1163/156854298X00084

[B53] Mohamed M. A. Coppel H. C. (1987). Pheromonal basis for aggregation behavior of parasitoids of the gypsy moth: *Brachymeria intermedia*, (Nees) and *Brachymeria lasus*, (Walker) (Hymenoptera: Chalcididae). *J. Chem. Ecol.* 13 1385–1393. 10.1007/BF01012285 24302240

[B54] Moreau S. J. M. Asgari S. (2015). Venom proteins from parasitoid wasps and their biological functions. *Toxins* 7 2385–2412. 10.3390/toxins7072385 26131769 PMC4516919

[B55] Parisi M. Nuttall R. Edwards P. Minor J. Naiman D. Lü J. (2004). A survey of ovary-, testis-, and soma-biased gene expression in *Drosophila melanogaster* adults. *Genome Biol.* 5:R40. 10.1186/gb-2004-5-6-r40 15186491 PMC463073

[B56] Parra J. R. P. Zucchi A. R. (2004). Trichogramma in Brazil: feasibility of use after twenty years of research. *Neotrop. Entomol.* 33 271–281. 10.1590/S1519-566X2004000300001

[B57] Persson B. Bray J. E. Bruford E. Dellaporta S. L. Favia A. D. Duarte R. G. (2008). The sdr (short-chain dehydrogenase/reductase and related enzymes) nomenclature initiative. *Chem. Biol. Interact.* 178 94–98. 10.1016/j.cbi.2008.10.040 19027726 PMC2896744

[B58] Pointer M. A. Harrison P. W. Wright A. E. Mank J. E. (2013). Masculinization of gene expression is associated with exaggeration of male sexual dimorphism. *PLoS Genet.* 9:e1003697. 10.1371/journal.pgen.1003697 23966876 PMC3744414

[B59] Popescu D. C. Ham A. J. Shieh B. H. (2006). Scaffolding protein INAD regulates deactivation of vision by promoting phosphorylation of transient receptor potential by eye protein kinase C in *Drosophila*. *J. Neurosci.* 26 8570–8577. 10.1523/JNEUROSCI.1478-06.2006 16914683 PMC1577681

[B60] Price D. Bell H. Hinchliffe G. Fitches E. Weaver R. Gatehouse J. A. (2009). Venom metalloproteinase from the parasitic wasp *Eulophus pennicornis* is toxic towards its host, tomato moth (*Lacanobia oleracae*). *Insect Mol. Biol.* 18 195–202. 10.1111/j.1365-2583.2009.00864.x 19320760

[B61] Prince E. G. Kirkland D. Demuth J. P. (2010). Hyperexpression of the X chromosome in both sexes results in extensive female bias of X-linked genes in the flour beetle. *Genome Biol. Evol.* 2 336–346. 10.1093/gbe/evq024 20624738 PMC2942036

[B62] Ranz J. Castillo-Davis C. Meiklejohn C. Hartl D. (2003). Sex-dependent gene expression and evolution of the Drosophila transcriptome. *Science* 300 1742–1745. 10.1126/science.1085881 12805547

[B63] Rinn J. L. Snyder M. (2005). Sexual dimorphism in mammalian gene expression. *Trends Genet.* 21 298–305. 10.1016/j.tig.2005.03.005 15851067

[B64] Simser D. H. Coppel H. C. (1980). Female-produced sex pheromone in *Brachymeria lasus* and *B. intermedia* (Hym.: Chalcididae). *BioControl* 25 373–380.

[B65] Spradling A. C. (1993). “Developmental genetics of oogenesis,” in *The Development of Drosophila*, eds Bate M. Martinez-Arias A. (Cold Spring Harbor, NY: Cold Spring Harbor Laboratory Press), 1–70.

[B66] Terayama M. (1999). “Description of new species of the family Bethylidae from the Ryukyus, and taxonomic notes on the Japanese species of the genus Sclerodermus,” in *Identification guide to the Aculeata of the Nansei Islands*, eds Seiki Y. Ikudome S. Terayama M. (Sapporo: Hokkaido University Press).

[B67] Tian C. Wang L. Ye G. Zhu S. (2010). Inhibition of melanization by a Nasonia defensin-like peptide: implications for host immune suppression. *J. Insect Physiol.* 56 1857–1862. 10.1016/j.jinsphys.2010.08.004 20708012

[B68] Wang T. Montell C. (2007). Phototransduction and retinal degeneration in *Drosophila*. *Pflügers Arch. Eur. J. Physiol.* 454 821–847. 10.1007/s00424-007-0251-1 17487503

[B69] Wang X. Werren J. H. Clark A. G. (2015). Genetic and epigenetic architecture of sex-biased expression in the jewel wasps *Nasonia vitripennis* and *giraulti*. *Proc. Natl. Acad. Sci. U.S.A.* 112 E3545–E3554. 10.1073/pnas.1510338112 26100871 PMC4500217

[B70] Wen X. Guo L. Jiao X. Yang N. Xin Y. Wu Q. (2014). Transcriptomic dissection of sexual differences in *Bemisia tabaci*, an invasive agricultural pest worldwide. *Sci. Rep.* 4:4088. 10.1038/srep04088 24526031 PMC3924218

[B71] Werren J. H. Richards S. Desjardins C. A. Niehuis O. Gadau J. Colbourne J. K. (2010). Functional and evolutionary insights from the genomes of three parasitoid *Nasonia* species. *Science* 327 343–348. 10.1126/science.1178028 20075255 PMC2849982

[B72] Wolff T. Ready D. (1993). “Pattern formation in the Drosophila retina,” in *The Development of Drosophila melanogaster*, eds Bate M. Arias A. M. (Plainview, NY: Cold Spring Harbor Lab. Press), 1277.

[B73] Yan J. J. Xu C. H. Li G. W. Zhang P. Y. Gao W. C. Yao D. F. (1989). *Parasites and Predators of Forest Pest.* Beijing: China Forestry Publishing House.

[B74] Yang X. Q. Wei J. R. Yang Z. Q. (2001). A survey on insect natural enemies of Hyphantriacunea in Dalian district, Liaoning Province. *Chin. J. Biol. Control* 17 40–42.

[B75] Young M. D. Wakefield M. J. Smyth G. K. Oshlack A. (2010). Gene ontology analysis for rna-seq: accounting for selection bias. *Genome Biol.* 11:R14. 10.1186/gb-2010-11-2-r14 20132535 PMC2872874

[B76] Zhishan W. Hopper K. R. Ode P. J. Fuester R. W. Jia-Hua C. Heimpel G. E. (2003). Complementary sex determination in Hymenopteran parasitoids and its implications for biological control. *Entomol. Sin.* 10 81–93. 10.1111/j.1744-7917.2003.tb00369.x

[B77] Zuker C. S. (1996). The biology of vision in *Drosophila*. *Proc. Natl. Acad. Sci. U.S.A.* 93 571–576. 10.1073/pnas.93.2.571 8570597 PMC40093

