# Supplementary material for: Sexual Transcription Differences in Brachymeria lasus (Hymenoptera: Chalcididae), a Pupal Parasitoid Species of Lymantria dispar (Lepidoptera: Lymantriidae)
Source: Front Genet. 2019 Mar 5;10:172. doi: 10.3389/fgene.2019.00172 (PMC6411638; doi:10.3389/fgene.2019.00172)
Supplement: Supplementary file 1 [file Data_Sheet_1.ZIP › Supplementary/Figure S1.docx]

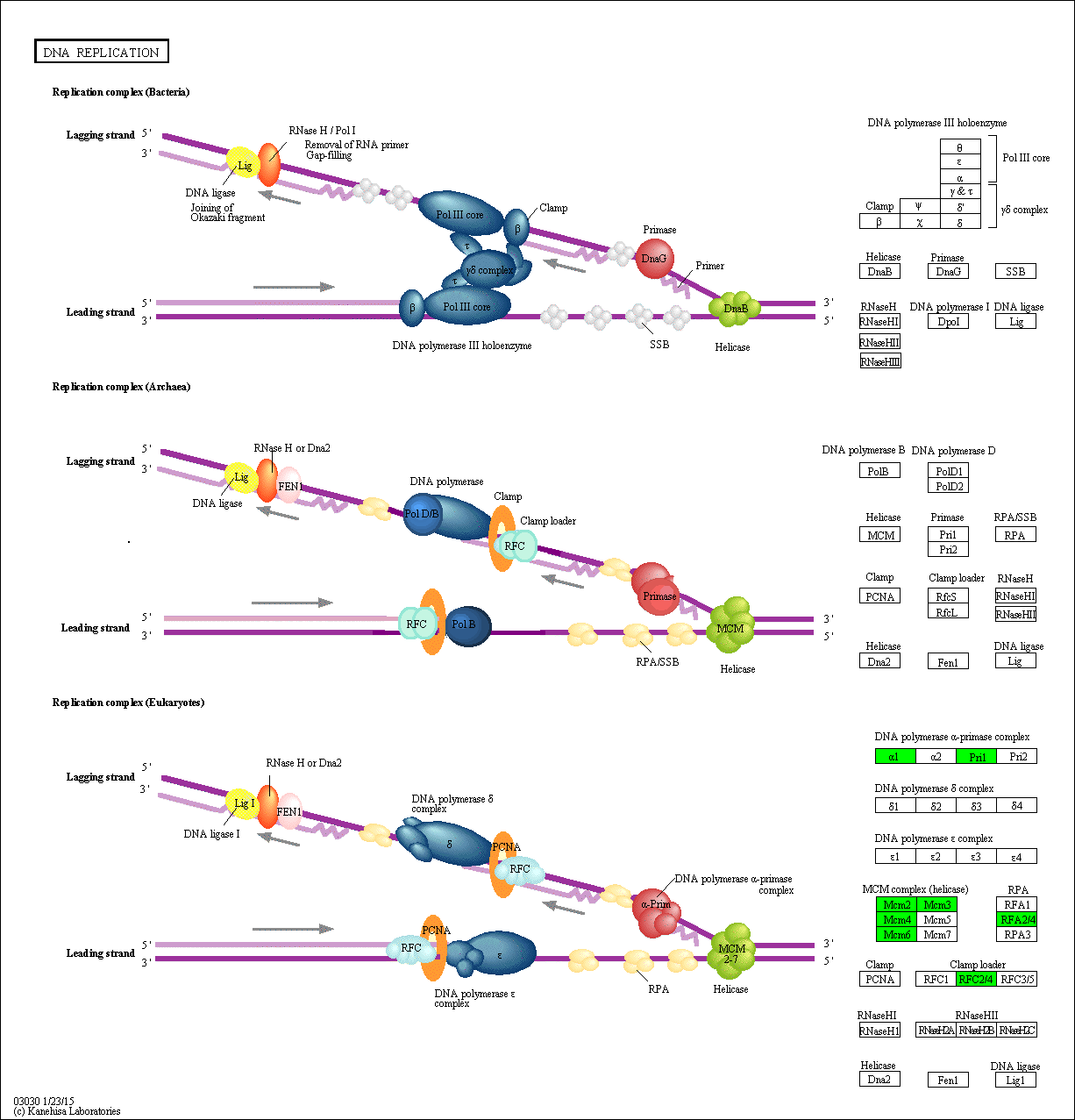


**Figure S1-a.** The enriched KEGG pathway of DNA replication


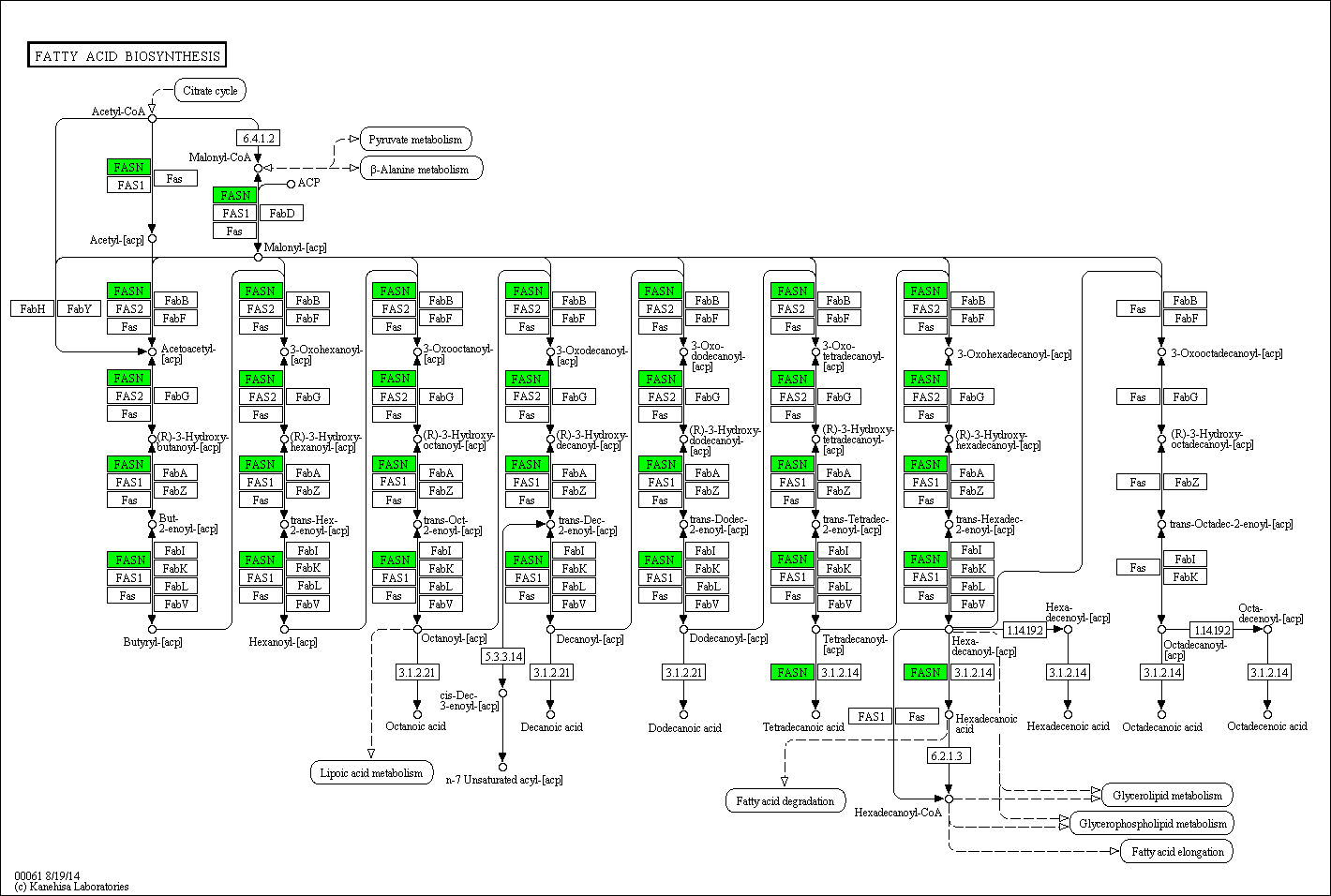


**Figure S1-b.** The enriched KEGG pathway of fatty acid biosynthesis


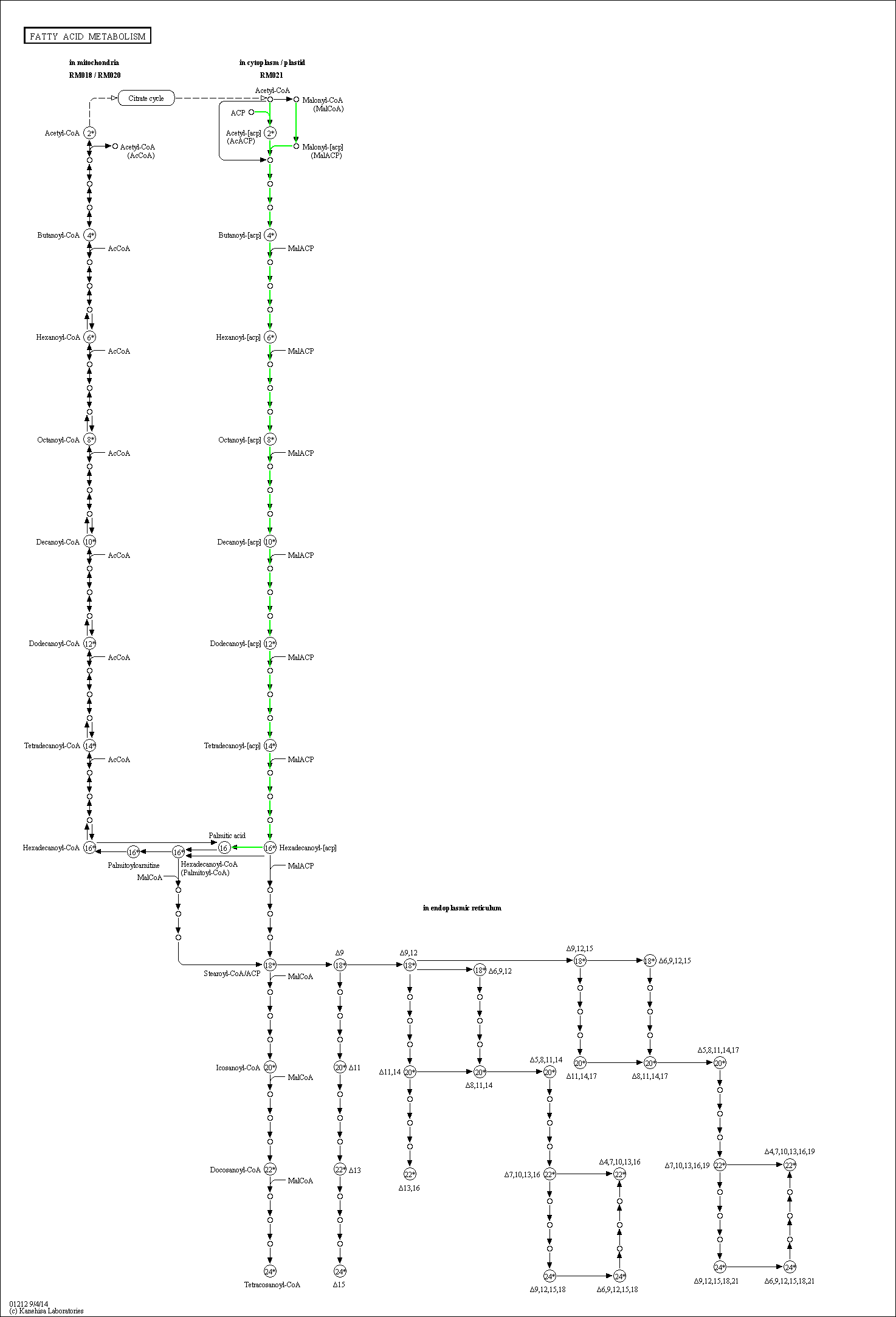


**Figure S1-c.** The enriched KEGG pathway of fatty acid metabolism.


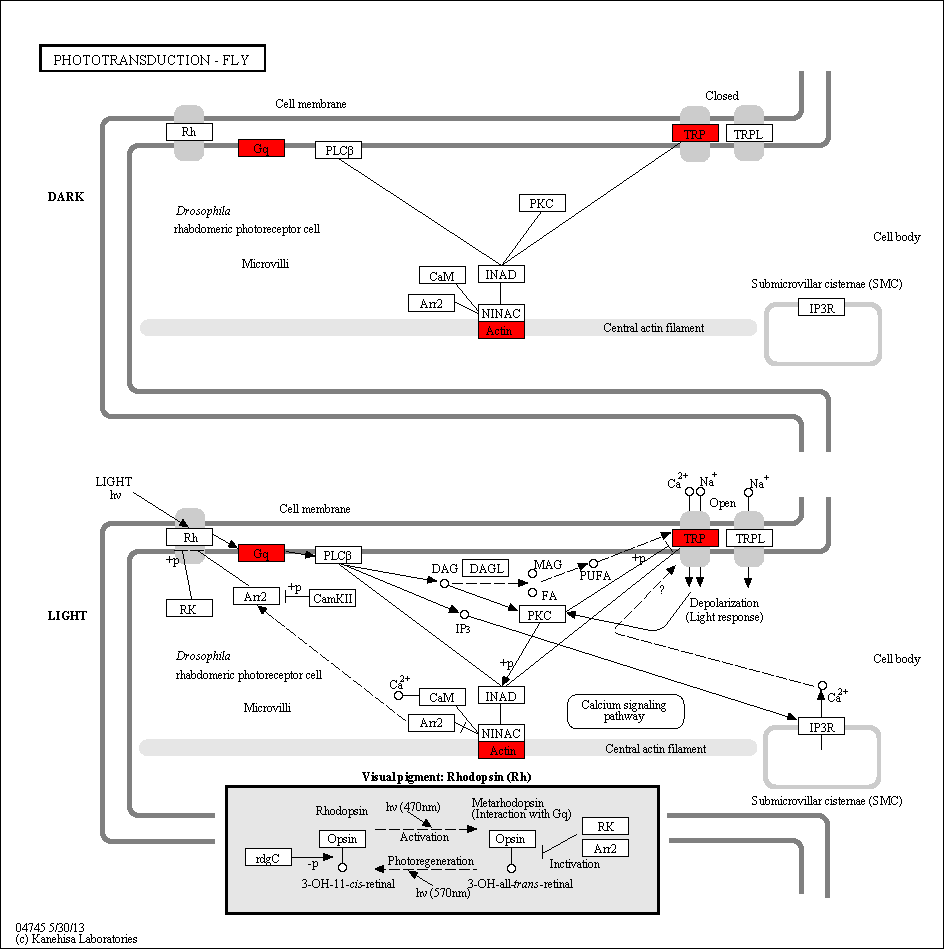


**Figure S1-d.** The enriched KEGG pathway of a phototransduction-fly.
