# Supplementary material for: Sexual Transcription Differences in Brachymeria lasus (Hymenoptera: Chalcididae), a Pupal Parasitoid Species of Lymantria dispar (Lepidoptera: Lymantriidae)
Source: Front Genet. 2019 Mar 5;10:172. doi: 10.3389/fgene.2019.00172 (PMC6411638; doi:10.3389/fgene.2019.00172)
Supplement: Supplementary file 1 [file Data_Sheet_1.ZIP › Supplementary/Table S2.docx]

**Table S2.** Statistics of transcriptome assembly and predicted unigenes

| Variable | Total number of assembled transcripts | Number of predicted unigenes |
| --- | --- | --- |
| Number | 254,656 | 164,709 |
| Size of data (bp) | 255,604,087 | 94,227,117 |
| N50 length (bp) | 2,706 | 814 |
| Mean length (bp) | 1,003.72 | 572.08 |
